# Abnormal cortical morphology in children and adolescents with intermittent exotropia

**DOI:** 10.3389/fnins.2022.923213

**Published:** 2022-10-04

**Authors:** Xi Wang, Lu Lu, Meng Liao, Hong Wei, Xiaohang Chen, Xiaoqi Huang, Longqian Liu, Qiyong Gong

**Affiliations:** ^1^Department of Ophthalmology, West China Hospital, Sichuan University, Chengdu, China; ^2^Laboratory of Optometry and Vision Sciences, West China Hospital, Sichuan University, Chengdu, China; ^3^Huaxi MR Research Center (HMRRC), Department of Radiology, West China Hospital, Sichuan University, Chengdu, China

**Keywords:** intermittent exotropia, magnetic resonance imaging, cortical morphology, surface-based morphometry, structural covariance

## Abstract

**Purpose:**

To investigate cortical differences, age-related cortical differences, and structural covariance differences between children with intermittent exotropia (IXT) and healthy controls (HCs) using high-resolution magnetic resonance imaging (MRI).

**Methods:**

Sixteen IXT patients and 16 HCs underwent MRI using a 3-T MR scanner. FreeSurfer software was used to obtain measures of cortical volume, thickness, and surface area. Group differences in cortical thickness, volume and surface area were examined using a general linear model with intracranial volume (ICV), age and sex as covariates. Then, the age-related cortical differences between the two groups and structural covariance in abnormal morphometric changes were examined.

**Results:**

Compared to HCs, IXT patients demonstrated significantly decreased surface area in the left primary visual cortex (PVC), and increased surface area in the left inferior temporal cortex (ITC). We also found increased cortical thickness in the left orbitofrontal cortex (OFC), right middle temporal cortex (MT), and right inferior frontal cortex (IFC). No significant differences were found in cortical volume between the two groups. There were several negative correlations between neuroanatomic measurements and age in the HC group that were not observed in the IXT group. In addition, we identified altered patterns of structural correlations across brain regions in patients with IXT.

**Conclusion:**

To our knowledge, this study is the first to characterize the cortical morphometry of the children and adolescents with IXT. Based on our results, children and adolescents with IXT exhibited significant alterations in the PVC and association cortices, different cortical morphometric development patterns, and disrupted structural covariance across brain regions.

## Introduction

Strabismus, the misalignment of the visual axes of the two eyes, is a visual disorder in humans preventing normal binocular vision (Friedman et al., 2009). Intermittent exotropia (IXT) is the most prevalent form of strabismus in some populations and usually develops in children and adolescents (Yu et al., 2002; McKean-Cowdin et al., 2013). Its incidence ranges from 1 to 3.9% in different regions (Govindan et al., 2005; Chia et al., 2010; Fu et al., 2014). IXT is characterized by an intermittent outward deviation of one eye, and it can result in binocular vision abnormalities (i.e., suppression, fusion and stereopsis impairment, and imbalanced ocular dominance) (Serrano-Pedraza et al., 2011b; Economides et al., 2012; Feng et al., 2015; Lavrich, 2015), which derive from abnormal binocular interactions originating at the cortical level (Serrano-Pedraza et al., 2011a). Indeed, these abnormalities may persistently exist even though the two eyes have been straightened by surgeries (Saunders and Trivedi, 2008; Feng et al., 2015; Wang et al., 2019). These phenomena may suggest that the certain regions in the brain may be affected by this special visual disorder.

Previous studies have detected brain morphological changes in strabismus animal models. The number of binocular neurons in the V1 region, the connections between V1 areas, and neuronal activity in ocular dominance columns of the striate cortex were reduced in these studies (Crawford et al., 1984; Sengpiel et al., 1994; Tychsen and Burkhalter, 1997; Fenstemaker et al., 2001; Toporova et al., 2006; Adams et al., 2013). Modern technologies for mapping the human brain allow investigators to explore the properties of the cortical structure associated with this visual disorder. While an increasing amount of evidence for brain functional changes has been found in patients with exotropia (Shi et al., 2019; Chen et al., 2021; He et al., 2021a) limited research has been conducted on the effects of exotropia on brain structure. With the structural MRI, Yin et al. (2021) found that patients with concomitant strabismus showed increased cortical thickness in the precentral gyrus and angular gyrus, and decreased cortical thickness in the frontal, parietal, occipital, and temporal cortex regions. Chan et al. (2004) used voxel-based morphometry (VBM) to analyze changes in gray and white matter volume in 10 exotropic adults compared with healthy controls (HCs). They found that the gray matter volume in the primary and extrastriate visual cortices was smaller in those with exotropia, while the gray matter volume in regions related to oculomotor was larger in those with exotropia. Yan et al. (2010) reported that there were structural abnormalities in occipital and parietal areas in adult patients with comitant exotropia using VBM and voxel-based analysis of diffusion tensor imaging. However, most of these prior neuroimaging studies were carried out with adult subjects, and few study has explored the cortical alterations in children or adolescents who are at the age of exotropia onset. In addition, cortical morphometric changes in IXT remain unknown.

Surface-based morphometry (SBM) techniques using non-linear alignment to cortical folding patterns, provide more accurate determinations of differences in brain volume (Ghosh et al., 2010; Gupta et al., 2019). This technique has been used in studying neuroanatomical changes in patients with abnormal visual disorders, such as monocular enucleation and amblyopia, helping investigators uncover the problems underlying these diseases (Kelly et al., 2015; Qi et al., 2016; Lu et al., 2019). In addition, structural covariance, a relatively novel measurement derived from the analysis of structural magnetic resonance imaging (MRI), can be used to characterize network-level brain features. With the help of this brain connectivity measurement, investigators can study the differences in regional volumes coordinated within brain networks that vary together in size (Evans, 2013). In postmortem studies, it has been reported that structural correlations exist in the human visual system in healthy individuals (Andrews et al., 1997). Recently, altered patterns of structural correlation across brain regions have been identified in those with strabismic amblyopia (Lu et al., 2019). However, whether IXT, which seldom leads to amblyopia (Lavrich, 2015), disrupts the normal structural covariance patterns has not yet been addressed.

In the current study, we used SBM to measure cortical surface area, thickness, and volume in a group of children and adolescents who experienced IXT and compared their results to a healthy control (HC) group. We also assessed age-related cortical differences and structural covariance differences between the two groups.

## Materials and methods

### Participants

This study was approved by the Research Ethics Committee of West China Hospital of Sichuan University, and written informed consent was obtained from all participants’ parents/guardians. A total of 16 IXT patients and 16 age-, sex-, handedness-, and education-matched HCs were recruited in this study. All subjects underwent an ophthalmologic examination, including visual acuity, cycloplegic refraction, slit-lamp biomicroscopy, fundus exam, prism and alternate cover test measuring angle of deviation, the Butterfly Stereo Acuity test, and the Newcastle Control Score to grade the severity of IXT (a higher score indicates a more severe IXT) (Lavrich, 2015). The diagnosis of IXT was independently made by two experienced ophthalmologists (LL, XW). The following exclusion criteria were used: (1) amblyopia; (2) a history of strabismus treatment; (3) any other ocular, neurologic or psychiatric disorders; (4) excessive head motion; and (5) serious systemic illness or MR imaging contraindications.

### Magnetic resonance imaging data acquisition and data processing

All participants were examined using the same 3-T MR scanner (Trio; Siemens, Erlangen, Germany) with an eight-channel phased-array head coil. Scanner noise was attenuated with earplugs, and head motion was restricted with foam padding around the head. High-resolution anatomical MRI was obtained with a spoiled gradient recalled sequence with the following parameters: repetition time/echo time, 2,250 ms/2.62 ms; flip angle, 9^°^; section thickness, 1 mm; sagittal plane of acquisition, 192 slices; no slice gaps; field of view, 256 × 256 mm^2^; and voxel size, 1 × 1 × 1 mm^3^.

For each participant, cortical thickness was estimated using the FreeSurfer pipeline (version of 5.3.0)^1^ at each vertex over the entire cerebral cortex, a process that has been validated against histological analysis on postmortem brains and manual measurements (Rosas et al., 2002; Salat et al., 2004). In brief, image analysis included visual inspection of the data for motion artifacts, transformation to Talairach space, intensity normalization, skull stripping, segmentation of subcortical white matter and gray matter structures (Fischl et al., 2002, 2004), tessellation of gray matter and white matter boundaries, and automated topology correction and surface deformation following intensity gradients to optimally place the gray/white and gray/cerebrospinal fluid borders defined at the location with the greatest shift in signal intensity (Dale et al., 1999; Fischl and Dale, 2000; Fischl et al., 2001; Segonne et al., 2007). Cortical volumes were measured based on cortical parcellations that considered cortical folding patterns (Fischl et al., 2004). Cortical thickness was quantified at each vertex as the distance (in mm) from the gray/white boundary to the pial surface (Fischl and Dale, 2000). Vertex-level cortical volume, thickness and surface area were obtained and mapped onto the normalized cortical surface and then smoothed with a 10-mm full-width at half-maximum kernel to improve the signal-to-noise ratio of the measurements.

### Statistical analysis

QDEC software^2^ was used to assess group differences in cortical volume, cortical thickness, and surface area at vertex level, using a general linear model with intracranial volume (ICV) and age as covariates. A Monte Carlo simulation with 10,000 iterations was used to control for multiple comparisons. The significance level was set at *P* < 0.05.

To test for potential differences in the structural covariance in brain regions between groups, we first extracted the averaged data from regions with significant group differences for each subject. Then we performed Pearson partial correlation analyses to identify the structural covariance among the regions that showed abnormal morphometric changes in the IXT group, controlling for ICV and age. Fisher Z-transformation was used to directly compare the correlation coefficients of the two groups.

## Results

The mean age of the 16 patients (13 men) in the IXT group was 11.23 ± 2 years. The subjects in the HC group were matched for age (11.86 ± 2.2 years; *p* = 0.397) and sex (11 men; *p* = 0.685) with the IXT patients. The detailed clinical characteristics of the two group are showed in Table 1. The visual parameters and demographic data of each individual with IXT are listed in Supplementary Table 1.

**TABLE 1:** Demographics and clinical measurements of IXT and HC groups.

Variables	IXT group	HC group	*P*-value
Age (years)	11.23 ± 2	11.86 ± 2.2	0.397
Sex (male/female)	13/3	11/5	0.685
Education (years)	5.23 ± 2	5.86 ± 2.2	0.397
NCS	5.12 ± 2	N/A	N/A
Near deviation (PD)	–41.75 ± 14	N/A	N/A
Distance deviation (PD)	–30.44 ± 11	N/A	N/A

Data are presented as mean ± standard deviation; IXT, intermittent exotropia; HC, healthy controls; NCS, Newcastle Score; PD, Prism diopter; N/A, not applicable.

### Group differences in cortical morphology

**FIGURE 1:**
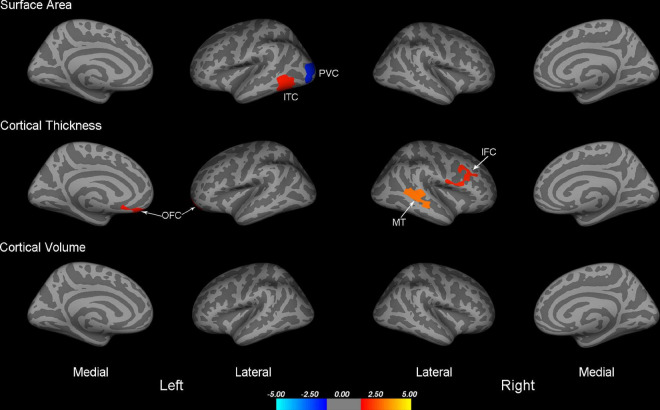
Maps of surface area, cortical thickness, and cortical volume differences between IXT and HC (*P* < 0.05, Monte Carlo corrected). PVC, primary visual cortex; ITC, inferior temporal cortex; OFC, orbitofrontal cortex; MT, middle temporal cortex; IFC, inferior frontal cortex; IXT, intermittent exotropia; HC, healthy controls.

Compared to HCs, IXT patients demonstrated significantly decreased surface area in the left primary visual cortex (PVC), and increased surface area in the left inferior temporal cortex (ITC) (Table 2 and Figure 1). We also found increased cortical thickness in the left orbitofrontal cortex (OFC), right middle temporal cortex (MT), and right inferior frontal cortex (IFC) (Table 2 and Figure 1). No significant differences were found in cortical volume between the two groups.

**TABLE 2:** Brain regions with altered surface area and cortical thickness in IXT compared to HC.

Measure	Hemisphere	Brain region	Talairach coordinates			Cluster size (mm^2^)	–log10(*p*)
			
			X	Y	Z		
**Surface area**							
IXT < HC	L	Primary visual cortex	–34.1	–76.8	10.0	1382.9	–2.197
IXT > HC	L	Inferior temporal cortex	–52.9	–57.2	–3.5	1333.8	2.995
**Cortical thickness**							
IXT > HC	L	Orbitofrontal cortex	–15.9	45.1	–14.9	837.5	3.018
IXT > HC	R	Middle temporal cortex	44.0	–36.8	5.3	1223.1	7.333
IXT > HC	R	Inferior frontal cortex	36.5	19.5	19.3	935.4	3.580

IXT, intermittent exotropia; HC, healthy controls; L, left hemisphere; R, right hemisphere. Negative values of –log10(*p*) represent decreased surface area and cortical thickness in intermittent exotropia.

### Group differences in age-related cortical morphometric characteristics

**FIGURE 2:**
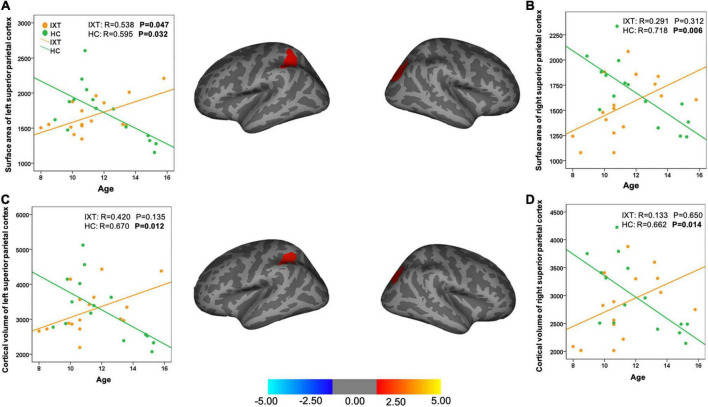
Brain maps and scatter plots showing statistically significant area-age **(A,B)** and volume-age **(C,D)** correlation differences between IXT patients and HC. IXT, intermittent exotropia; HC, healthy controls.

We found the relationship between age and the cortical morphometrics were different between patients and controls (Table 3 and Figure 2). Specifically, age was negatively correlated with surface area and cortical volume in the left and the right superior parietal cortex (SPC) in HC. However, in the patients with IXT, age was positively correlated only with surface area in the left SPC (*r* = 0.538, *p* = 0.047) (Table 3 and Figure 2A). No significant correlations between age and the other morphometric measures in the SPC were observed in this group (Figures 2B–D).

**TABLE 3:** Brain regions with altered age-related cortical morphometric measures in IXT compared to HC.

Measure	Hemisphere	Brain region	Talairach coordinates			Cluster size (mm^2^)	–log10(p)
			
			X	Y	Z		
**Surface are**							
IXT > HC	L	Superior parietal cortex	–45.7	–39.6	39.9	1226.3	4.256
IXT > HC	R	Superior parietal cortex	24.6	–78.0	24.2	1317.3	3.340
**Cortical volume**							
IXT > HC	L	Superior parietal cortex	–29.9	–46.1	45.7	922.9	4.211
IXT > HC	R	Superior parietal cortex	34.1	–71.0	23.6	920.4	2.915

IXT, intermittent exotropia; HC, healthy controls; L, left hemisphere; R, right hemisphere. Negative values of –log10(p) represent cortical thinning in intermittent exotropia.

### Structural covariance analysis

**FIGURE 3:**
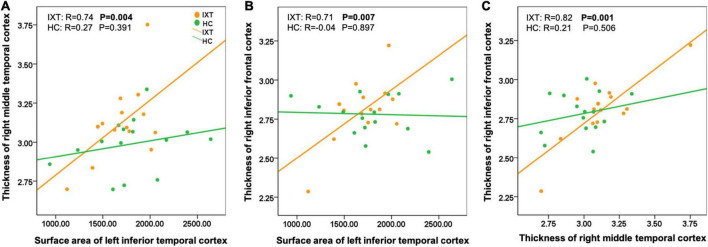
Scatter plots showing structural covariance differences between patient with IXT and HC **(A–C)**. IXT, intermittent exotropia; HC, healthy controls.

Among regions with significant morphometric alterations in the IXT group, there were significantly positive correlations between the following 3 pairs of regions: (1) cortical thickness of the right MT and surface area of the left ITC (*r* = 0.74, *p* = 0.004; Figure 3A); (2) cortical thickness of the right IFC and surface area of the left ITC (*r* = 0.71, *p* = 0.007; Figure 3B); and (3) cortical thickness of the right IFC and cortical thickness of right MT (*r* = 0.82, *p* = 0.001; Figure 3C). In contrast, non-significant correlations were found in the HC group. Direct statistical comparisons of correlation coefficients between the two groups exhibited significant differences between each pair of regions described above (*p* < 0.05).

### Clinical correlations analysis

There were no significant correlations between strabismic characteristics and any identified cerebral morphometric abnormalities in the IXT group.

## Discussion

This study indicates for the first time that children and adolescents with IXT had morphological changes in the visual cortex and associated cortices using high-resolution MRI. In addition, our results indicated that IXT relative to HC showed a lack of normative age-related reduction and structural covariance in surface area and cortical volume in the specific regions.

In the current study, we found significant reduction in surface area in the left PVC in IXT patients. This observation is consistent with morphological studies of strabismic animals. Previous studies found that the number of binocular neurons in the V1 regions was reduced in cats and monkeys with strabismus (Crawford et al., 1984; Sengpiel et al., 1994; Fenstemaker et al., 2001). Abnormal ocular dominance column structure of the striate cortex (Tychsen and Burkhalter, 1997) and changes in connections of columns of fields 17 and 18 were detected (Toporova et al., 2006; Schmidt and Lowel, 2008). Further biochemical studies reported that a reduction was observed in neuronal activity was observed in ocular dominance columns of the striate cortex of monkeys raised with exotropia (Adams et al., 2013). In the human research, accumulating evidences from functional MRI indicates that patients with strabismus are associated with abnormal brain functions. It has been reported that patients with concomitant exotropia had functional connections changes between the PVC and other visual related regions. Zhu et al. (2018) and He et al. (2021a) reported that patients with IXT exhibited significantly lower functional connectivity between the right V1 and the right calcarine sulcus, suggesting an impairment integrations within the V1 of IXT. Our study, focusing on cortical morphology of IXT, is partly consistent with previous structural MRI studies of humans with exotropia. Yin et al. (2021) found that children with concomitant strabismus not only showed a decreased cortical thickness in parieto-occipital sulcus but also in other regions including left intraparietal sulcus, superior and middle temporal gyrus, right ventral premotor cortex, anterior insula, OFC, and paracentral lobule. This wider range of cortical impairments may be due to the study including different types of strabismic. Chan et al. (2004) found with VBM that the gray matter volume in exotropic adults was smaller in V1. However, their study also observed reduced gray matter in extrastriate regions, posterior intraparietal sulcus, and right inferior parietal lobule, but no other significant reductions in morphometric measures were found in our study. Divergent findings are not unexpected, give that subjects in their study were adults (age: 22–40 years old) and they had amblyopia, a neurodevelopmental disorder that can lead to structural changes in visual portions of the brain (Mendola et al., 2005). In contrast, the mean age of our subjects was 11 years of age (range: 6–16), which is around the onset of IXT. Therefore, our study offered an opportunity to better explore the neurological mechanisms of strabismus. To our knowledge, there have been no prior neuroimaging studies of children with strabismus. Additionally, IXT patients in our study had normal corrected visual acuity, which can exclude the potential influence of amblyopia.

An interesting phenomenon was that our data found increased surface area and cortical thickness in visual association cortices. The abnormal function and structure of these visual association cortices were previously reported to be associated with strabismus. Increased regional homogeneity values in ITC (Huang et al., 2016), increased amplitude of low-frequency fluctuation in the prefrontal cortex and superior temporal gyrus (Yin et al., 2021), increased functional connectivity within MT area (He et al., 2021b) were found in the exotropic patients. The ITC may correspond to the visual area V4, a component of the ventral stream that plays a role in object perception (Schoenfeld et al., 2002; Freud et al., 2016). MT (also known as V5) is a subdivision of the dorsal stream that has a crucial role in object localization and visually guided action (Freud et al., 2016). The ventral and dorsal pathways have been discovered to be connected (Borra et al., 2010), and were found to project to shared regions in the frontal cortex (Distler et al., 1993; O’Reilly, 2010; Yeterian et al., 2012; Cloutman, 2013). A possible explanation for the increases in surface area and cortical thickness in the IXT group is a disruption in synaptic pruning during development due to abnormal binocular vision input to visual cortical networks. Exotropic animal models that exhibited disruptions in binocular synaptic integration (Scholl et al., 2013) may provide support for this interpretation. Further studies with larger samples are needed to address this.

Developmental differences in morphometric measures in specific regions between the IXT and HC groups were observed in the current investigation. Compared with HCs, those with IXT exhibited a lack of normal age-related reduction in the surface area and cortical volume in the SPC. Previous developmental studies of maturational trajectories of HCs have shown that synaptic density is higher than the adult level during early postnatal brain development, which is followed by a process of synaptic remodeling and pruning during childhood and adolescence (Petanjek et al., 2011; Hogstrom et al., 2013). The cellular or molecular underpinnings of neuroimaging features cannot be directly inferred, but decreases in cortical thickness and gray matter may reflect synaptic pruning (Giedd et al., 1999). Our results exhibited altered developmental trajectories in cortical thickness and volume in the IXT group relative to the HC group, suggesting age-related brain structural dysmaturation in children and adolescents with IXT. Although the underlying mechanisms of brain dysmaturation in IXT are unknown, we postulate that these abnormalities may be connected with abnormal visual experiences of those with IXT that affect normal cortical thinning. However, age-related changes cannot be accurately assessed by cross-sectional studies, and longitudinal studies are required to make this claim.

In this study, we also found abnormal structural covariance patterns in patients with IXT. To our knowledge, the structural covariance in regional brain anatomy has yet to be investigated in exotropia. The meaning of this kind of structural MRI analysis appears to reflect developmental coordination or synchronized maturation between regions of the brain, with potential value in understanding various neurological conditions (Alexander-Bloch et al., 2013). Our results identified that differences in synchronized anatomical organization across brain regions were observed in IXT patients compared with HCs. The disruption of visual information processing in the visual cortex of IXT patients and the persistence of this deficit may account for these altered patterns of structural correlations.

Potential limitations in our study should be mentioned. First, the sample size in this study although it is comparable to that in the previous investigations also focusing on children strabismus, was not very large. Larger sample MRI studies may help better understand in explicit changes in brain structure and correlations between the cortical abnormalities and clinical characteristics. Thus, further research is needed to confirm the generalizability of our findings. Second, the current study is somewhat limited by the cross-sectional design, although we closely matched the subjects on sex, education, and age. Longitudinal research (Guo et al., 2022) can help explore the dynamic changes of brain morphology of IXT to reveal its cortical mechanism of brain development and treatment responsiveness. Third, considering the cooperation during testing, children under 6 years were not recruited, and therefore, data from these early years are lacking.

## Conclusion

In conclusion, our study detected morphologic changes in the visual cortex and association cortices of human subjects with IXT. In addition, we also found different cortical morphometric development patterns in the IXT group. The normal structural covariance observed in the HCs was disrupted in the patients with IXT. These findings suggest possible disruptions in cortical visual networks and the cortical maturation resulting from this special visual disorder.

## Data availability statement

The original contributions presented in this study are included in the article/Supplementary material, further inquiries can be directed to the corresponding author/s.

## Ethics statement

The studies involving human participants were reviewed and approved by the Ethics Committee of West China Hospital of Sichuan University. Written informed consent to participate in this study was provided by the participants’ legal guardian/next of kin.

## Author contributions

XW, LL, XH, and LqL contributed to conception and design of the study. XH and QG provided technological support. ML, HW, and XC contacted patients to collect data. XW, LL, and ML performed the statistical analysis. XW and LL wrote the first draft of the manuscript. All authors contributed to the manuscript revision and approved to the submission.

## Footnotes

1
http://ftp.nmr.mgh.harvard.edu/

2
https://surfer.nmr.mgh.harvard.edu/fswiki/Qdec
