# Supplementary material for: Abnormal cortical morphology in children and adolescents with intermittent exotropia
Source: Front Neurosci. 2022 Oct 4;16:923213. doi: 10.3389/fnins.2022.923213 (PMC9577327; doi:10.3389/fnins.2022.923213)
Supplement: Supplementary file 1 [file Table_1.docx]

**Table S1.** Demographic and clinical characteristics of patients with intermittent exotropia.

| Subject | Age | Sex | NCS | Stereopsis | VA (logMAR) | | Refraction Status | | Exodeviation (PD) | |
| --- | --- | --- | --- | --- | --- | --- | --- | --- | --- | --- |
|  |  |  |  |  | OD | OS | OD | OS | Near | Distance |
| S1 | 10.0 | M | 3 | 100” | 0 | 0 | -3.75/-0.50×180 | +1.25×90 | -25 | -20 |
| S2 | 10.6 | M | 5 | 100” | 0 | 0 | -3.50 | -1.00 | -35 | -25 |
| S3 | 13.4 | M | 8 | 160” | 0 | 0 | -3.50 | -2.25/-0.50×175 | -80 | -50 |
| S4 | 10.6 | M | 7 | 200” | 0 | 0 | +0.50/+1.00×95 | +0.50/-1.00×180 | -35 | -20 |
| S5 | 8.5 | M | 5 | 63” | 0 | 0 | -1.50 | -1.25 | -35 | -20 |
| S6 | 13.6 | M | 4 | 63” | 0 | 0 | -1.00/-1.00×180 | -0.50/-1.00×180 | -50 | -35 |
| S7 | 13.2 | M | 4 | 20” | 0.08 | 0 | -2.25 | +0.75×90 | -45 | -35 |
| S8 | 15.8 | M | 3 | 160” | 0 | 0 | -0.50/-1.50×10 | +0.75/-2.00×170 | -50 | -35 |
| S9 | 10.6 | F | 3 | 32” | 0 | 0 | -4.75 | -4.00/-0.50×180 | -35 | -22 |
| S10 | 10.1 | M | 8 | N | 0 | 0 | -0.50 | -1.25 | -40 | -40 |
| S11 | 10.6 | M | 4 | 25” | 0 | 0 | +3.00/+0.50×65 | +3.00/+1.50×75 | -30 | -18 |
| S12 | 12.0 | F | 4 | 100” | 0 | 0 | -6.50/-3.00×180 | -4.75/-3.50×180 | -28 | -17 |
| S13 | 8.0 | F | 4 | 25” | 0 | 0 | PL | PL | -30 | -35 |
| S14 | 11.2 | M | 8 | 100” | 0 | 0 | -4.75/-1.50×10 | -5.00/-1.50×170 | -60 | -40 |
| S15 | 11.5 | M | 4 | 50” | 0 | 0 | -1.00×170 | -3.00/-2.50×180 | -40 | -25 |
| S16 | 9.9 | M | 8 | N | 0 | 0 | -2.50×170 | -0.50/-1.50×180 | -50 | -50 |

NCS, Newcastle Score; VA, Visual acuity; logMAR, Logarithm of minimum angle of resolution; PD, Prism diopter; OD, oculus dexter; OS, oculus sinister; N, no; PL, plano lens.
